# Collagen mineralization with lepidocrocite *via* Fe(OH)_2_ addition†

**DOI:** 10.1039/d1ce01527c

**Published:** 2022-01-21

**Authors:** Bernette M. Oosterlaken, Mark M. J. van Rijt, Heiner Friedrich, Gijsbertus de With

**Affiliations:** Laboratory of Physical Chemistry, Department of Chemical Engineering and Chemistry, Eindhoven University of Technology PO Box 513 5600 MB Eindhoven The Netherlands b.m.oosterlaken@tue.nl G.deWith@tue.nl; Center for Multiscale Electron Microscopy, Eindhoven University of Technology PO Box 513 5600 MB Eindhoven The Netherlands; Institute for Complex Molecular Systems, Eindhoven University of Technology PO Box 513 5600 MB Eindhoven The Netherlands

## Abstract

The mineralization of collagen *in vitro* has been extensively investigated for hydroxyapatite, silica, calcium carbonate and lepidocrocite (γ-FeOOH). Henceforth, it is interesting to investigate whether collagen also could serve as a generic mineralization template for other minerals, like magnetite. To this end, and inspired by the partial oxidation approach, first a ferrous hydroxide (Fe(OH)_2_) intermediate is synthesized *via* the titration of base to a solution of Fe^2+^. Subsequently, the Fe(OH)_2_ is mixed with collagen fibrils and poly(aspartic acid) is added to promote the formation of intrafibrillar crystals. Platelet-shaped lepidocrocite crystals being present throughout the entire thickness of the collagen fibrils can be realized, as was confirmed with electron tomography. The formation of lepidocrocite, which is an Fe^3+^ compound, is hypothesized to be induced *via* oxidation of the Fe^2+^ species and, therefore, the oxygen concentration during titration, TEM sample preparation and drying of TEM samples are investigated. Although the reaction is sensitive to small changes in experimental conditions, highly mineralized collagen fibers can be realized.

## Abstract 2

Intrafibrillar lepidocrocite platelets are shown to be present throughout the entire thickness of collagen fibrils using electron tomography.

## Graphical abstract

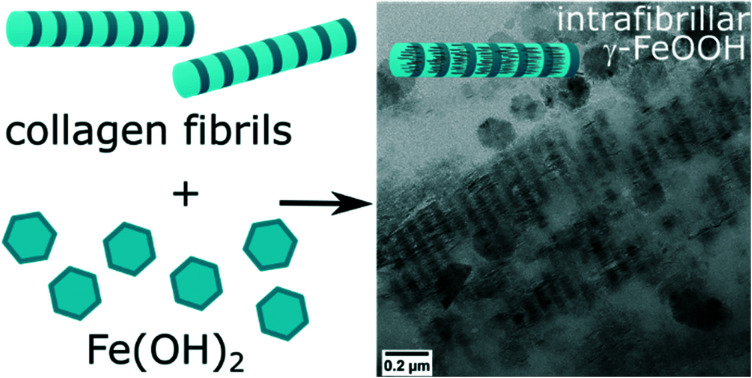

## 1. Introduction

Collagen, one of the most abundant proteins in nature, is known to mineralize *in vivo* with hydroxyapatite (HAp) in the bones and teeth of vertebrates. Mimicking the process of bone formation, collagen can be mineralized with HAp in synthetic procedures, in the presence of a charged polymer, like poly(aspartic acid) (pAsp).^1–3^ Presumably, the negatively charged polymer increases the barrier for nucleation in solution, thereby driving the formation of intrafibrillar mineral.^4^

Fundamentally, it is interesting to investigate whether collagen could serve as a generic mineralization template, noting that to this end, collagen has already been mineralized with calcium carbonate,^5–7^ silica,^8^ yttria–zirconia,^9^ lepidocrocite^10^ (γ-FeOOH) and iron hydroxide nanoparticles.^11^ Mineralization of collagen with magnetite is interesting as collagen–magnetite hybrids could be promising materials for bone cancer treatment^12,13^*via* magnetically induced hyperthermia that causes tumoral cell apoptosis, targeted (drug) delivery and for diagnosis using magnetic resonance imaging. However, although (partial) mineralization of collagen with various minerals has been achieved, many aspects of the process are still unclear.^3^

In our previous work,^11^ the mineralization of collagen *via* a coprecipitation method was described, leading to the formation of intrafibrillar iron(iii) hydroxide nanoparticles with a diameter of about 2.7 nm. As only small nanocrystals were obtained, we attempted another well-known bio-inspired synthesis route towards magnetite, namely partial oxidation. The partial oxidation approach starts with the synthesis of ferrous hydroxide *via* the addition of a base to a solution of Fe^2+^. Then, the Fe(OH)_2_ is partially oxidized to magnetite *via* a green rust (GR) intermediate. Acidic (bio)polymers are known to influence the magnetite crystal size and morphology.^14,15^

Here, first a solution of Fe^2+^ is titrated with KOH to induce the formation of ferrous hydroxide. The resulting crystals are added to dispersed collagen fibrils and similar to other collagen mineralization procedures, pAsp, supposedly acting as an intrafibrillar crystallization promoter, is added.^3,4^ Following this procedure, collagen could be mineralized with lepidocrocite. Although the system is sensitive to the experimental conditions, highly mineralized fibrils can be obtained. To the best of our knowledge, the partial oxidation approach has not been attempted before in connection to collagen.

## 2. Materials & methods

### 2.1. Materials

Ferrous chloride tetrahydrate (FeCl_2_·4H_2_O), potassium hydroxide pellets, HCl solution (ACS reagent, 37%) and poly(aspartic acid) (pAsp, poly-(α,β)-dl-aspartic acid sodium salt, *M*_w_ 2000–11 000) were purchased from Sigma Aldrich. Resorbable collagen tapes (RCT resorbable collagen tape, 2.5 × 7.5 cm^2^, bovine collagen type I) were acquired from Henry Schein Dental. All reagents were used without further purification. Collagen tapes were crushed under liquid nitrogen before use. MilliQ water was de-aerated under argon flow for at least 1 h and subsequently under nitrogen flow for another 15 min. All solutions and dispersions were prepared using de-aerated MilliQ water.

### 2.2. Collagen mineralization

Mineralization of collagen was performed inside a wet MBraun MB 200B glovebox under nitrogen atmosphere ([O_2_] < 5 ppm, unless stated otherwise). Titration experiments were performed at room temperature with a Metrohm Titrando 901 automated titration set-up, controlled by a computer running the software program Tiamo 2.5, and equipped with a glass pH electrode (Metrohm article number 6.0234.100), a Dosino 10 mL dosing device (KOH) and a Dosino 2 mL dosing device (HCl).

For the standard mineralization procedure, first 0.17 mmol pAsp was mixed with 1 mL collagen at a concentration of 5 mg mL^−1^ in de-aerated MilliQ water and left standing for approximately 15 min. Then, 0.05 mmol Fe(ii)Cl_2_·4H_2_O is dissolved in 3.85 mL de-aerated MilliQ water. The solution was titrated with 0.7 M KOH at a titration rate of 0.01 mL min^−1^ until pH 9 was reached. The resulting blue dispersion was added to the collagen–pAsp mixture and the pH of the mixture was adjusted to 8.5 *via* the titration of 0.5 M HCl at a titration rate of 0.01 mL min^−1^ without further degassing. The mixture was left to stir for 72 h or 2 weeks, after which a TEM sample was prepared.

In an alternative approach, collagen (1 mg mL^−1^), pAsp (34 mM) and FeCl_2_·4H_2_O (10 mM) were mixed in 4.85 mL MilliQ water. The mixture was titrated with 0.7 M KOH at a titration rate of 0.01 mL min^−1^ until pH 9 is reached. The mixture was left to stir for 72 h.

### 2.3. TEM, cryo-TEM and electron tomography

TEM grids, continuous carbon 200 mesh gold support, were surface plasma treated for 40 s using a Cressington 208 carbon coater prior to use. A sample volume of 20 μL was deposited on a TEM grid and left to dry on filter paper inside the wet MBraun glovebox under nitrogen atmosphere, typically for more than 2 h, unless stated otherwise. TEM imaging was performed on a Tecnai T20 G2 (Thermo Fisher Scientific), operating at 200 kV and equipped with an LaB_6_ filament. The images were acquired on a 4k × 4k CETA CMOS camera (Thermo Fisher Scientific).

For electron tomography, TEM samples were prepared as described above. The grids were back-labelled with 10 nm Au fiducials to facilitate alignment of the tilt-series. Back-labelling of the grids was performed by placing a TEM grid on top of a droplet of an Au particle dispersion on parafilm for 1 min, with the back side of the grid facing the droplet. Subsequently, the grid was washed by placing it on top of a MilliQ droplet for 1 min and another 20 s on a second MilliQ droplet, followed by drying. The tilt series was collected between −66° and +66° using 3° increments. The total electron dose for tilt-series acquisition was 135 e^−^ Å^−2^. The tilt series was acquired using Inspect3D software (Thermo Fisher Scientific) and aligned and reconstructed using IMOD software using the Simultaneous Iterative Reconstructive Technique (SIRT) algorithm.

The drying experiments were performed by applying 2 μL of sample to a surface plasma treated TEM grid that was held by an inverted tweezer. The tweezer was placed above a Petri dish filled with water inside the glovebox. To minimize evaporation and to create a ∼100% RH atmosphere, a second Petri dish was placed to cover the sample. For the experiments at RH = 40%, the tweezer was left inside the glovebox, in which the RH is constant at about 40%. In both cases care was taken that neither the tweezer nor the grid was touching any surface and the samples were left untouched until they were fully dry. As the grids were densely covered with solids, the grids were washed by placing it on top of a MilliQ droplet for 1 min, with the sample facing the droplet. Washing was finalized by placing the grid on another MilliQ droplet for 1 min and 20 s on a third droplet, followed by drying.

Cryo-TEM samples were prepared on 200 mesh gold support holey carbon grids. The TEM grids were surface plasma treated for 40 s using a Cressington 208 carbon coater prior to use. A 3 μL sample was applied to a TEM grid and blotted for 3.5 s in an automated vitrification robot (Thermo Fisher Scientific Vitrobot Mark III). As it is important to prevent oxidation of the intermediates and/or products during cryo-TEM sample preparation and analysis, the Vitrobot is directly connected to the wet MBraun glovebox. Both glovebox and Vitrobot were customized with an airlock and flange, respectively, that allows direct access between glovebox and vitrification chamber.^11^

Cryo-TEM imaging was performed on the TU/e cryo-TITAN TEM (Thermo Fisher Scientific) operated at 300 kV and equipped with a field emission gun (FEG), a post column Gatan 2002 energy filter (GIF) and a post-GIF 2k × 2k Gatan model 794 CCD camera. The images were acquired with an electron dose of 7 e^−^ Å^−2^ per image.

## 3. Results

**Fig. 1:**
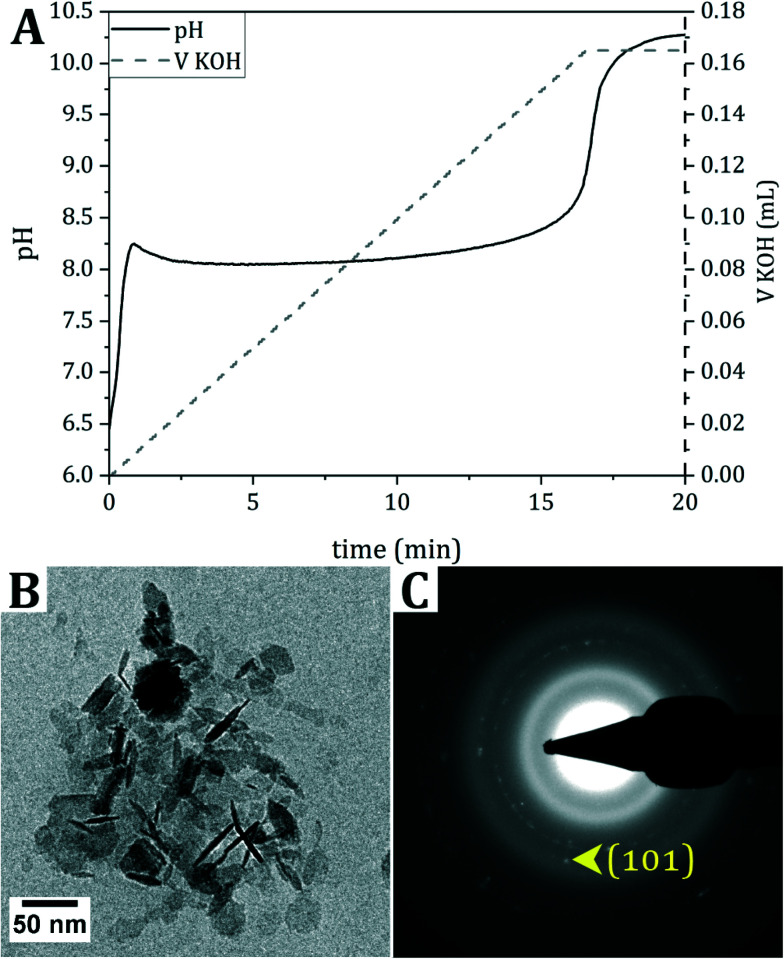
Titration of Fe^2+^. A. Evolution of pH upon the continuous addition of 0.7 M KOH as a base to a solution of FeCl_2_. B. Cryo-TEM of the product directly after titration of a solution of FeCl_2_. C. Selected area electron diffraction of the product in B, matching δ-FeOOH or Fe(OH)_2_. The characteristic (101) signal, distinguishing δ-FeOOH from Fe(OH)_2_, is indicated.

For the mineralization of collagen, first pAsp, presumed to promote intrafibrillar collagen mineralization,^3,4^ was mixed with dispersed collagen fibrils. Subsequently, a solution of Fe^2+^ in water was titrated with 0.7 M KOH, while continuously monitoring the pH until pH 9 was reached (Fig. 1A). To prevent oxidation of the reactants and products, titration was performed inside a glovebox under N_2_ atmosphere. The product was analyzed with cryogenic transmission electron microscopy (cryo-TEM) (Fig. 1B and C and S1†).

During the titration of KOH, the pH immediately rises until pH 8.2 is reached. Then, the pH evolves into a plateau value, indicating the consumption of OH^−^ ions. Finally, the pH increases again to the set value of 9, generally overshooting to pH 10, at which point base titration is stopped.

The cryo-TEM image of the product directly after titration indicates the formation of hexagonal crystals, and selected area electron diffraction (SAED) points towards δ-FeOOH. However, δ-FeOOH is nearly indistinguishable from Fe(OH)_2_ (Table S1†). Based on the reaction conditions and the blueish color of the dispersion after titration,^16^ it is hypothesized that Fe(OH)_2_ forms during the reaction, which, despite all precautions taken to prevent oxidation during vitrification, (partially) oxidizes into δ-FeOOH during cryo-TEM sample preparation.

**Fig. 2:**
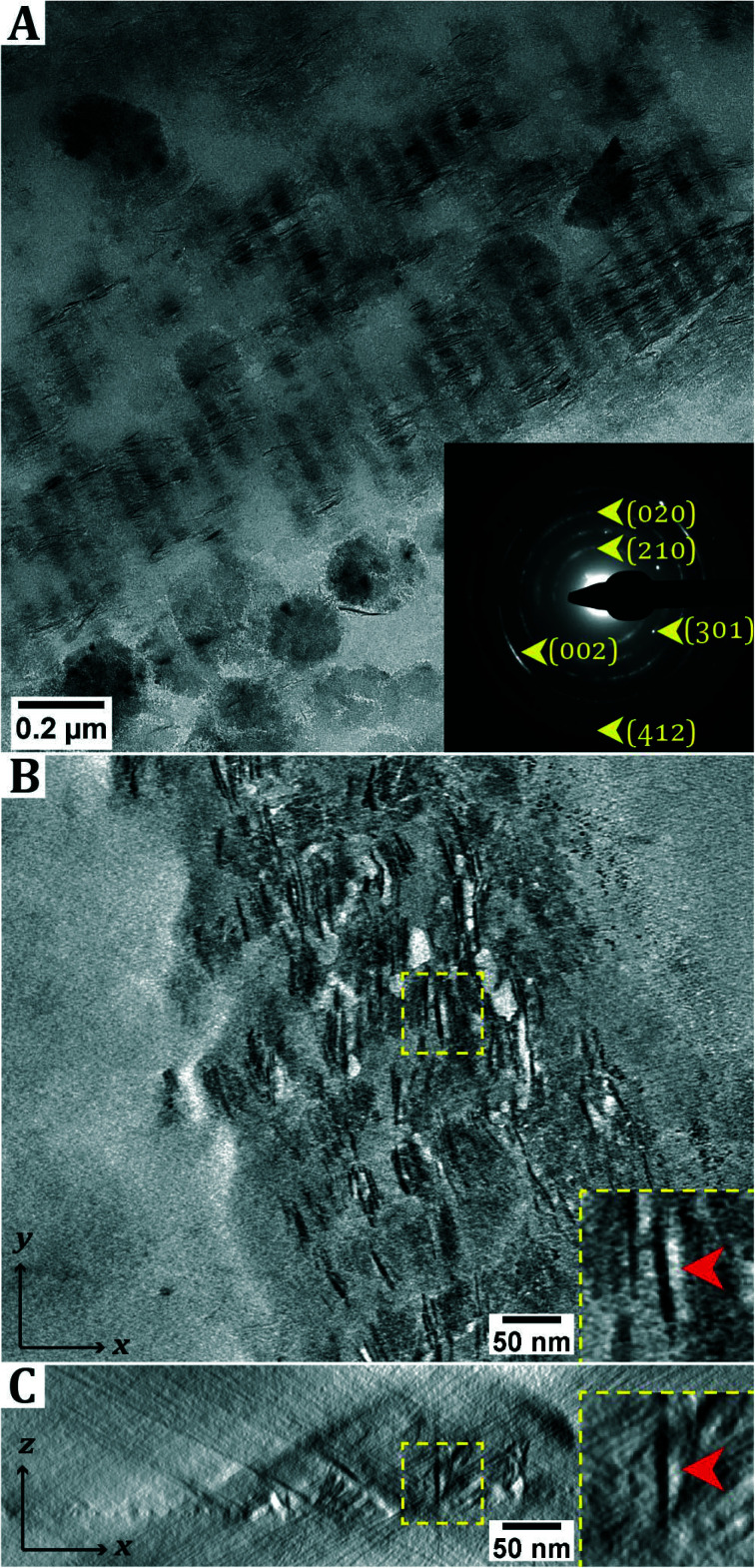
Collagen mineralization *via* the addition of Fe(OH)_2_. A. Dry-TEM image of a mineralized collagen fibril, which was mineralized by adding Fe(OH)_2_ to a dispersion of collagen fibers and pAsp. The sample was aged for two weeks. Inset: SAED of the image in a indicates the formation of lepidocrocite, with the (002) aligned with the collagen. B and C. Electron tomography results of collagen fibrils mineralized *via* addition of Fe(OH)_2_ crystals. B. Numerical cross-section (thickness: 2.3 nm) through the reconstructed volume along the *xy*-plane. C. Numerical cross-section (thickness: 2.3 nm) through the reconstructed volume along the *xz*-plane, corresponding to same crystals as shown in image B. Inset: Magnified image of the crystal in the 3D-reconstructed volume. Arrows indicate the same crystal in both slices. Images are averaged over 3 slices to reduce noise.

Subsequently, the synthesized Fe(OH)_2_ was added to the collagen–pAsp mixture, having a pH of 8.5, and the pH of the resulting dispersion was adjusted to 8.5 *via* titration with 0.5 M HCl (Fig. S2†). After aging the reaction solution for 72 h, samples were taken (Fig. S3†) and after continued aging of the reaction solution for two weeks, another set of samples was taken. Both sets of samples were prepared for analysis with TEM in the dry state (hereafter: ‘dry-TEM’) (Fig. 2A).

In dry-TEM, mineralized collagen fibrils were observed after aging the sample for two weeks. The SAED pattern matches that of lepidocrocite. The observation of arcs in the diffraction pattern indicates that the crystals are aligned with the (002) axis parallel to the collagen. Outside the collagen green rust (type I, Fe^II^_*x*_Fe^III^_*y*_OH_2*x*+3*y*–*z*_Cl_*z*_) crystals were found in cryo-TEM (Fig. S4†), which is consistent with the observation that the solution darkens from light blue to green upon aging. Similar to the product obtained directly after titration, δ-FeOOH is observed in dry-TEM, which is possibly due to oxidation of GR upon exposure to air. From the 2D projection as given by conventional TEM, it is not apparent whether the crystals are inside or outside the collagen fibril. Moreover, from these images it cannot be determined whether the crystals are needle-shaped or platelets that are viewed edge-on. To resolve this question, electron tomography (ET) was performed (Fig. 2B and C; ESI† section 2).

When analyzing the three-dimensional (3D) reconstruction, it became clear that platelet-shaped lepidocrocite crystals are present throughout the entire thickness of the collagen fibril, though it should be noted that the fibrils are relatively thin (<100 nm).

**Fig. 3:**
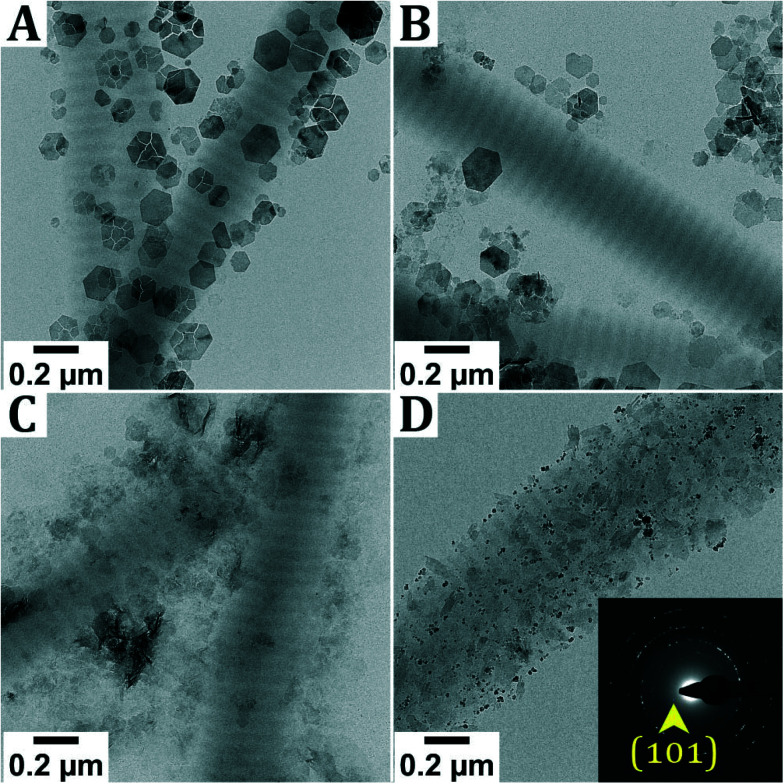
Performing the mineralization reaction at different concentrations of oxygen. Dry-TEM images of reactions performed in: A. [O_2_] = 2 ppm (glovebox), B. [O_2_] = 40 ppm (glovebox), C. [O_2_] = 170 ppm (glovebox) and D. [O_2_] = 21% (laboratory environment). In images A and C δ-FeOOH is identified. In D the formation of magnetite is observed, alongside goethite. Due to overlapping signals, the SAED is not assigned, except for the characteristic (101) signal for goethite.

These results appeared to be challenging to reproduce, indicating that the system is highly sensitive to the reaction conditions. As the formation of lepidocrocite is often the result of oxidation of Fe^2+^ species,^16^ the mineralization was performed at different oxygen concentrations inside the glovebox (Fig. 3A and C) and in air (Fig. 3D). As the need for the pH adjustment to pH 8.5 to obtain highly mineralized fibers was not directly apparent, as highly mineralized fibers were also obtained without this pH adjustment (Fig. S5†), this step was not performed for these screening experiments.

Performing the experiment at different oxygen concentrations inside the glovebox does not lead to significant differences in the obtained reaction product. In all cases, hexagonal crystals are observed next to the collagen. As described earlier, it is hypothesized that these are Fe(OH)_2_ or GR, oxidized into δ-FeOOH during sample transfer. Only when the reaction is performed in air, different products are observed, namely magnetite and goethite (α-FeOOH) crystals, which are only found outside the collagen. Furthermore, when performing the titration inside the glovebox but leaving the reaction dispersion in air while aging, no intrafibrillar mineralization was observed (Fig. S6†).

The results above indicate that oxidation towards lepidocrocite does not take place in solution, but rather at a different moment, for example during TEM sample preparation. Furthermore, mineralized fibrils were almost exclusively observed next to larger aggregates of collagen fibrils, indicating that drying effects and concomitant spatial concentration gradients may play a role. To investigate this, a variety of TEM samples was prepared following different drying procedures.

**Fig. 4:**
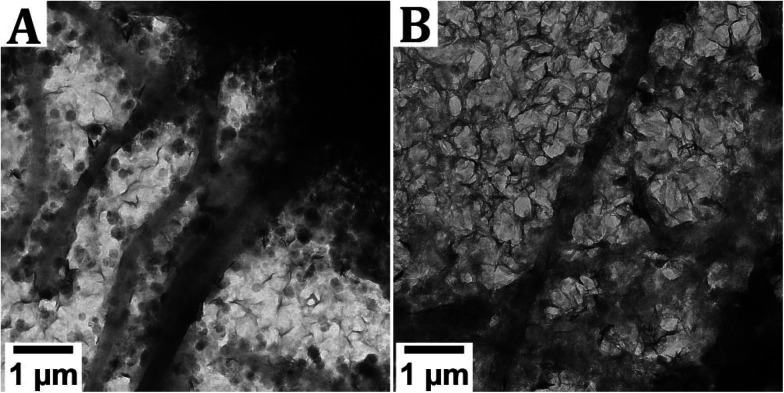
The effect of relative humidity on the product during drying of the TEM grid. For both grids, a sample that appeared mineralized previously was used (Fig. 2). The grids were dried in A. 40 % RH and B. near-100% RH.

First, TEM samples were prepared following the standard drying method, in which a TEM grid is placed on top of a filter paper and a 20 μL droplet is deposited on top of the grid. These samples were dried inside the glovebox, which has a relative humidity (RH) of 40%. The samples were removed from the glovebox after different drying times (Fig. S7†), but no lepidocrocite formation was observed. Following a different approach, the sample was applied as a 2 μL droplet to a TEM grid and left to dry in 40% RH (Fig. 4A) and in near-100% RH (Fig. 4B).

When drying the sample in 40% RH, the formation of hexagonal crystals is observed, along with sheet-like material. The hexagonal crystals are either Fe(OH)_2_ or GR, oxidized to δ-FeOOH. The sheet-like material could not be identified, as it does not give a signal in SAED, but it is hypothesized that these sheets are an iron-pAsp precipitate. Conversely, in the sample that was dried in a near-100% RH, the predominant phase is the sheet-like material, while occasionally also hexagonal crystals can be distinguished. For both samples, the grid was densely covered with reaction products and no mineralized collagen fibrils were observed.

Finally, doubling the pAsp concentration or doubling both the pAsp and the Fe concentration did not result in essentially different results, and control experiments using the same conditions and concentrations but in the absence of pAsp resulted, as expected, only in hexagonal GR crystals grown outside the collagen (Fig. S8†).

## 4. Discussion

This paper describes the mineralization of collagen following a partial oxidation-inspired approach. The results of all mineralization reactions are summarized in Table 1.

**Table 1:** Summary of the results from different mineralization reactions

Experimental description	Reaction conditions		Drying conditions		Results		Fig.
	Final pH	[O_2_] (ppm)	[O_2_] (ppm)	RH^a^ (%)	Intrafibrillar mineralization^b^ (yes/no)	Iron species^c^	
Titration of FeCl_2_	10	5	5	40	n. a.	δ-FeOOH (Fe(OH)_2_)	1 and S1†
Addition of Fe(OH)_2_ to collagen and pAsp	8.5	5	5	40	No (72 h)	δ-FeOOH (Fe(OH)_2_)	S3†
Addition of Fe(OH)_2_ to collagen and pAsp	8.5	5	5	40	Yes (2 weeks)	γ-FeOOH (inside)	2 and S4†
						GR (outside)	
Addition of Fe(OH)_2_ to collagen and pAsp	9^d^	5	5	40	Yes (72 h)	γ-FeOOH (inside)	S5†
						GR (outside)	
Addition of Fe(OH)_2_ to collagen and pAsp	9^d^	2	5	40	No (1 week)	GR	3A
Addition of Fe(OH)_2_ to collagen and pAsp	9^d^	40	5	40	No (1 week)	GR	3B
Addition of Fe(OH)_2_ to collagen and pAsp	9^d^	170	5	40	No (1 week)	GR	3C
Addition of Fe(OH)_2_ to collagen and pAsp	9^d^	20%	5	40	No (1 week)	G and M	3D
Addition of Fe(OH)_2_ to collagen and pAsp	8.5	5	5	40	No (>8 weeks)^e^	GR and pAsp–Fe	4A
Addition of Fe(OH)_2_ to collagen and pAsp	8.5	5	5	100	No (>8 weeks)^e^	pAsp–Fe and GR	4B
Addition of Fe(OH)_2_ to collagen and pAsp	9^d^	5	20%^f^	40	No (1 h)	GR and M	S6A†
Addition of Fe(OH)_2_ to collagen and pAsp	8.5	5	20%^f^	40	No (1 h)	GR and M	S6B†
Titration of collagen, pAsp and FeCl_2_	9	5	20%	40	No (>8 weeks)^g^	pAsp–Fe	S6C†
Addition of Fe(OH)_2_ to collagen and pAsp	8.5	5	20%	40	No (>8 weeks)^e^^,^^g^	GR	S6D†
Addition of Fe(OH)_2_ to collagen and pAsp	8.5	5	20%^h^	40	No (>8 weeks)^e^	GR	S7A†
Addition of Fe(OH)_2_ to collagen and pAsp	8.5	5	5	40	No (>8 weeks)^e^	GR	S7B†
Addition of Fe(OH)_2_ to collagen and pAsp^i^	8.5	5	5	40	No (2 weeks)	GR	S8A†
Addition of Fe(OH)_2_ to collagen and pAsp^j^	8.5	5	5	40	No (2 weeks)	GR and pAsp–Fe	S8B†
Addition of Fe(OH)_2_ to collagen^k^	8.5	5	5	40	No (72 h; 2 weeks)	GR	S8C and D†
Addition of Fe(OH)_2_ to collagen and pAsp	7.5	5	5	40	No (72 h)	pAsp–Fe	S9A†
Addition of Fe(OH)_2_ to collagen and pAsp	6	5	5	40	No (72 h)	No crystals	S9B†
Titration of collagen, pAsp and FeCl_2_	9	5	5	40	Maybe (72 h)	pAsp–Fe	S10†

a Only the RH during drying is reported, as all reactions were performed under 40% RH.

b In brackets the time between titration and preparation of the TEM sample is given (= ageing time).

c Iron species: the species observed in TEM are given, the species in brackets () denotes the species that was most likely present before drying/vitrifying the sample. Abbreviations: GR = green rust, G = goethite, M = magnetite and with pAsp–Fe we mean the sheetlike structures hypothesized to be pAsp–Fe complexes.

d No pH adjustment step was performed.

e No mineralization observed, even though the same dispersion resulted in mineralized fibers (Fig. 2).

f Freshly made dispersions aged in air for 1 h.

g Solution aged inside the glovebox first for several weeks, then exposed to air for 3 h.

h Dried inside the glovebox ([O_2_] = 5 ppm) for 5 min, then in air for another 30 min.

i Double pAsp concentration.

j Double pAsp and double Fe concentration.

k Without pAsp.

First, a solution of Fe^2+^ is titrated with base. The formation of ferrous hydroxide, Fe(OH)_2_, was expected, but δ-FeOOH was observed in the TEM sample instead. Based on the blueish color of the reaction solution and the fact that the SAED signals from Fe(OH)_2_ and δ-FeOOH are almost identical, it is hypothesized that Fe(OH)_2_ forms, which visibly oxidizes into orange δ-FeOOH upon exposure to air during cryo-TEM sample preparation or transfer of dry-TEM samples from the glovebox to the microscope.

After mixing the Fe(OH)_2_ crystals with collagen and pAsp, the formation of intrafibrillar lepidocrocite crystals was observed. The mineral phase is present throughout the entire thickness of the collagen fibril, similar to what we observed in our previous study.^11^ Different from our previous work, however, is that we observe mature, elongated lepidocrocite crystals inside the collagen in this work, whereas only nanoparticles of 2.7 nm in size were observed previously.

Our results match those obtained by Xu *et al.*^10^ in terms of the lepidocrocite crystals being aligned with the (002) axis along the collagen. However, Xu *et al.*^10^ observed lepidocrocite formation in the outer 25 nm of the collagen only, while here the lepidocrocite crystals are present throughout the entire thickness of the collagen fibril. Xu *et al.*^10^ attributed the mineralization in the outer rim to the limited solubility of the Fe^3+^ ions.^16^ However, in our system Fe^2+^ is the predominant species, suggesting that the formation of lepidocrocite is induced *via* a different mechanism.

Lepidocrocite often forms through the oxidation of Fe^2+^ at neutral to mildly acidic conditions (pH 6–7).^16^ More specifically, the formation of lepidocrocite from green rust typically occurs at low O_2_ levels, whereas high oxygen concentrations typically lead to the formation of magnetite.^17^ Indeed, when performing the reaction at high O_2_ concentrations (21%), the formation of magnetite and goethite was observed. Varying the O_2_ concentration between 2 ppm and 300 ppm inside the glovebox, however, had no effect on intrafibrillar mineralization and *δ*-FeOOH, possibly an oxidation product of Fe(OH)_2_ or GR, was found next to the collagen in all cases. In these experiments, the pH was not adjusted to 8.5, but in an experiment where the pH of the mixture of Fe(OH)_2_ crystals with collagen and pAsp was adjusted to pH 7.5, only the formation of extrafibrillar Fe(OH)_2_ was observed (Fig. S9†), which is assigned based on the color of the dispersion and not based on SAED.

Although all precautions were taken to prevent oxidation during titration and sample preparation, some oxidation cannot be ruled out. In fact, upon aging some oxidation is indeed observed, as the dispersion changes color from light blue to green, consistent with oxidation from Fe(OH)_2_ to GR. These results could indicate that in our system, either oxidation is not the main driving force for the formation of lepidocrocite inside collagen or that oxidation towards lepidocrocite is not occurring in dispersion, but, for example, during the preparation and transfer of TEM samples. Furthermore, it was observed that mineralized fibrils are almost exclusively found next to bigger aggregates of collagen fibrils, indicating that drying effects might play a role in mineral deposition inside the collagen. To verify that, the TEM sample preparation methods were varied.

Varying the drying time inside the glovebox of a TEM grid placed on top of a filter paper did not result in significant differences between the samples. The relative humidity in which the samples were dried, however, did have a limited effect on the observed product. Whereas hexagonal crystals and sheet-like material are present in a 50/50 ratio when the sample was dried in 40% RH, sheet-like crystals became the primary phase present when the sample was dried at close to 100% RH. The reason for this difference is yet unclear. The sheet-like crystals could not be identified but are hypothesized to be an iron-pAsp precipitate.

Based on previous experiments^11^ and the well-established knowledge that intrafibrillar HAp crystals are solely obtained when pAsp is added,^3^ it was hypothesized that pAsp is necessary to induce the formation of intrafibrillar crystals. Based on the control experiment in absence of pAsp (Fig. S8†), pAsp is indeed necessary to drive the formation of intrafibrillar crystals. Investigating the role of pAsp in more detail, an experiment was performed in which FeCl_2_ was mixed with pAsp and collagen prior to the addition of base. Upon titration until pH 9, the formation of sheet-like material next to the collagen was observed (Fig. S10†), which appears amorphous in SAED. This could indicate that pAsp binds some of the Fe^2+^ material to form amorphous sheets and prevents crystallization into Fe(OH)_2_ or GR, though it is not clear what the role of pAsp is in the mineralization reaction. Notably, the procedure presented here starts with a solid precursor Fe(OH)_2_ instead of a dissolved species as is normally the case in collagen mineralization procedures. This probably leads to a different mineralization mechanism. Possibly, somewhere in the transition from Fe(OH)_2_ to lepidocrocite, an intermediate phase is formed, which can enter the collagen and crystallize into elongated platelet-shaped crystals.

Thus, although highly mineralized fibrils can be obtained, the mechanism through which lepidocrocite forms inside the collagen is not yet established. Lepidocrocite might be favored over the thermodynamically more stable goethite in the presence of phosphate groups^18^ which could be present on the collagen. The formation of lepidocrocite might be templated by the collagen, as lepidocrocite also favors a layered, platelet-shaped morphology. However, as mineralization is only observed for a number of samples, templating effects alone are not sufficient to induce the formation of lepidocrocite and other factors are most likely at play. However, limited reproducibility obscures which specific factors influence the reaction pathway towards highly mineralized fibers.

In particular, as oxidative effects might induce the formation of lepidocrocite, the procedure presented here is specific to multivalent cation-based minerals, such as the iron oxides. Nevertheless, we have demonstrated that mineralization of collagen with platelet-shaped lepidocrocite is possible to a considerable degree. Thus, even though the reaction pathway might be specific for the system presented here, it was demonstrated that collagen could indeed serve as a generic mineralization template.

## 5. Conclusions

In this paper, the mineralization of collagen *via* a method inspired by the partial oxidation approach was investigated. First, a solution of Fe^2+^ was titrated with KOH, leading to the formation of Fe(OH)_2_, which oxidizes to δ-FeOOH upon exposure to air. The Fe(OH)_2_ crystals were added to a mixture of collagen and pAsp to induce the formation of intrafibrillar crystals. Formation of platelet-shaped lepidocrocite crystals throughout the thickness of the collagen fibrils was observed, with the (002) crystal axis aligned with the collagen.

Several parameters were tested for their influence on the mineralization reaction. Varying the oxygen concentration during the reaction does not lead to different products, except when the reaction is performed in air. The humidity in which the sample was dried seems to have some effect, as amorphous sheet-like material is observed instead of Fe(OH)_2_ under near-100% humidity.

Thus, although sensitive to the reaction conditions, highly mineralized fibrils with intrafibrillar platelet-shaped lepidocrocite crystals can be realized.

## Abbreviations

2D: Two-dimensional
3D: Three-dimensional
ET: Cryo-electron tomography
cryo-TEM: Cryo-transmission electron microscopy
GR: Green rust
pAsp: Poly(aspartic acid)
RH: Relative humidity
SAED: Selected area electron diffraction
TEM: Transmission electron microscopy

## Author contributions

The manuscript was written through contributions of all authors. All authors have given approval to the final version of the manuscript.

## Conflicts of interest

There are no conflicts to declare.

## Supplementary Material

CE-024-D1CE01527C-s001
