# Supplementary material for: Collagen mineralization with lepidocrocite via Fe(OH)2 addition
Source: CrystEngComm. 2022 Jan 21;24(6):1211–7. doi: 10.1039/d1ce01527c (PMC8819979; doi:10.1039/d1ce01527c)
Supplement: CE-024-D1CE01527C-s001 [file CE-024-D1CE01527C-s001.pdf]

Electronic Supplementary Material (ESI) for CrystEngComm.
This journal is © The Royal Society of Chemistry 2022

Electronic Supplementary Information

for

Collagen Mineralization with Lepidocrocite via $Fe(OH)_2$ Addition

authored by

Bernette M. Oosterlaken, Mark M. J. van Rijt, Heiner Friedrich and Gijsbertus de With

This electronic supplementary information consists of the following sections:

Section 1 – Experimental Section

Section 2 – Additional Images and Control Experiments

Section 3 – Electron Tomography

Section 1 – Experimental Section

S1.1. Materials

Ferrous chloride tetrahydrate ($FeCl_2.4H_2O$), potassium hydroxide pellets, HCl solution (ACS reagent, 37 %) and poly(aspartic acid) (pAsp, poly-(α,β)-DL-aspartic acid sodium salt, M_w 2000 – 11,000) were purchased from Sigma Aldrich. Resorbable collagen tapes (RCT resorbable collagen tape, 2.5 × 7.5 cm^2, Bovine Collagen type I) were acquired from Henry Schein Dental. All reagents were used without further purification. Collagen tapes were crushed under liquid nitrogen before use. MilliQ water was de-aerated under argon flow for at least 1 h and subsequently under nitrogen flow for another 15 min. All solutions and dispersions were prepared using de-aerated MilliQ water.

S1.2. Collagen Mineralization

Mineralization of collagen was performed inside a wet MBraun MB 200B glovebox under nitrogen atmosphere ($[O_2] < 5$ ppm, unless stated otherwise). Titration experiments were performed at room temperature with a Metrohm Titrando 901 automated titration set-up, controlled by a computer running the software program Tiamo 2.5, and equipped with a glass pH electrode (Metrohm article number 6.0234.100), a Dosino 10 mL dosing device (KOH) and a Dosino 2 mL dosing device (HCl).

For the standard mineralization procedure, first 0.17 mmol pAsp was mixed with 1 mL collagen at a concentration of 5 mg mL^{-1}. This is left standing for approximately 15 min. Then, 0.05 mmol $Fe(II)Cl_2 \cdot 4H_2O$ is dissolved in 3.85 mL de-aerated MilliQ water. The solution was titrated with 0.7 M KOH at a titration rate of 0.01 mL min^{-1} until pH 9 was reached. The resulting blue dispersion was added to the collagen-pAsp mixture and the pH of the mixture was adjusted to 8.5 via the titration of 0.5 M HCl at a titration rate of 0.01 mL min^{-1}. The mixture was left to stir for 72 h or 2 weeks, after which a TEM sample was prepared.

In an alternative approach, collagen (1 mg mL^{-1}), pAsp (34 mM) and $FeCl_2.4H_2O$ (10 mM) were mixed in 4.85 mL MilliQ water. The mixture was titrated with 0.7 M KOH at a titration rate of 0.01 mL min^{-1} until pH 9 is reached. The mixture was left to stir for 72 h.

S1.3. TEM, Cryo-TEM and Electron Tomography

TEM grids, continuous carbon 200 mesh gold support, were surface plasma treated for 40 s using a Cressington 208 carbon coater prior to use. A sample volume of 20 µL was deposited on a TEM grid and left to dry on filter paper inside the wet MBraun glovebox under nitrogen atmosphere, typically for more than 2 h, unless stated otherwise. TEM imaging was performed on a Tecnai T20 G2 (Thermo Fisher Scientific), operating at 200 kV and equipped with an LaB_6 filament. The images were acquired on a 4k × 4k CETA CMOS camera (Thermo Fisher Scientific).

For electron tomography, TEM samples were prepared as described above. The grids were back-labelled with 10 nm Au fiducials to facilitate alignment of the tilt-series. Back-labelling of the grids was performed by placing a TEM grid on top of a droplet of an Au particle dispersion on parafilm for 1 min, with the back side of the grid facing the droplet. Subsequently, the grid was washed by placing it on top of a MilliQ droplet for 1 min and another 20 s on a second MilliQ droplet, followed by drying. The tilt series was collected between −66° and +66° using 3° increments. The total electron dose for tilt-series acquisition was 135 e^{-} $Å^{-2}$. The tilt series was acquired using Inspect3D software (Thermo Fisher Scientific) and aligned and reconstructed using IMOD software using the Simultaneous Iterative Reconstructive Technique (SIRT) algorithm.

The drying experiments were performed by applying 2 µL of sample to a surface plasma treated TEM grid that was held by an inverted tweezer. The tweezer was placed above a Petri dish filled with water inside the glovebox. To minimize evaporation and to create a ~100 % RH atmosphere, a second Petri dish was placed to cover the sample. For the experiments at RH = 40%, the tweezer was left inside the glovebox, in which the RH is constant at about 40 %. In both cases care was taken that neither the tweezer nor the grid was touching any surface and the samples were left untouched until they were fully dry. As the grids were densely covered with solids, the grids were washed by placing it on top of a MilliQ droplet for 1 min, with the sample facing the droplet. Washing was finalized by placing the grid on another MilliQ droplet for 1 min and 20 s on a third droplet, followed by drying.

Cryo-TEM samples were prepared on 200 mesh gold support holey carbon grids. The TEM grids were surface plasma treated for 40 s using a Cressington 208 carbon coater prior to use. A 3 µL sample was applied to a TEM grid and blotted for 3.5 s in an automated vitrification robot (Thermo Fisher Scientific Vitrobot Mark III). As it is important to prevent oxidation of the intermediates and/or products during cryo-TEM sample preparation and analysis, the Vitrobot is directly connected to the wet MBraun glovebox. Both glovebox and Vitrobot were customized with an airlock and flange, respectively, that allows direct access between glovebox and vitrification chamber[1].

Cryo-TEM imaging was performed on the TU/e cryo-TITAN TEM (Thermo Fisher Scientific) operated at 300 kV and equipped with a field emission gun (FEG), a post column Gatan 2002 Energy Filter (GIF) and a post-GIF 2k × 2k Gatan model 794 CCD camera. The images were acquired with an electron dose of 7 e^{-} $Å^{-2}$ per image.

S2. Additional Images and Control Experiments

Table S1. Diffraction data as experimentally obtained (Figure 1, main text) compared to literature values. Literature values were taken from Cornell and Schwertmann[2].

Experimental distances (nm)	$Fe(OH)_2$ *d* (nm)	$Fe(OH)_2$ Crystal plane	δ-FeOOH *d* (nm)	δ-FeOOH Crystal plane
0.455	0.460	n.d. [a)]	0.461	(001)
0.272	0.282	n.d. [a)]	n.p. [b)]	n.p. [b)]
0.246	0.240	n.d. [a)]	0.255	(100)
0.219	n.p. [b)]	n.p. [b)]	0.223	(101)
0.174	0.178	n.d. [a)]	0.169	(102)
0.165	0.163	n.d. [a)]	0.169	(102)
0.151	0.154	(111) or (003)	0.147	(110)
0.144	0.135	(103) or (201)	0.127	(200)

[a)] n.d. = not determined. For $Fe(OH)_2$ many of the diffraction signals were not assigned to a crystal plane.
[b)] n.p. = not present. For $Fe(OH)_2$ there is no known signal around 0.22 nm. Similarly, for 0.27 nm, there is no matching signal for δ-FeOOH.

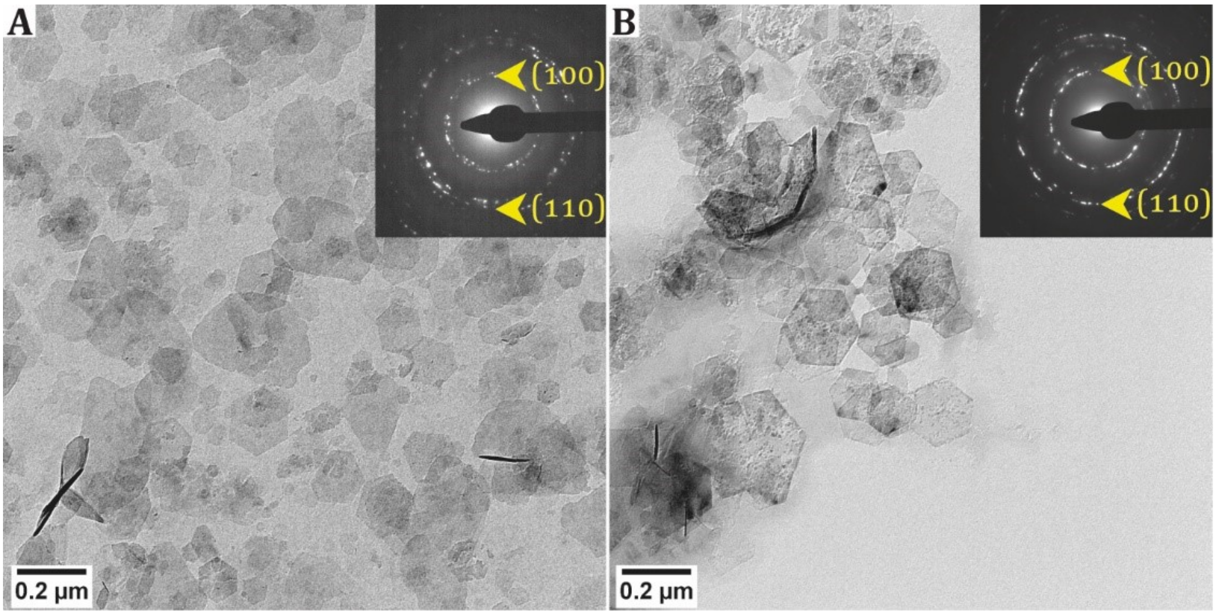

Figure S1. Product after titration of Fe^{2+}. A. Dry TEM image of the product at pH 9, in which δ-FeOOH is observed. **B.** Dry TEM image of the product after aging the reaction dispersion for 72 h, showing that the δ-FeOOH crystals are still present, although some appear damaged. Insets: SAED patterns corresponding to the TEM images. The most apparent signals are indicated and match δ-FeOOH, which probably is an oxidation product of $Fe(OH)_2$ upon exposure to air.

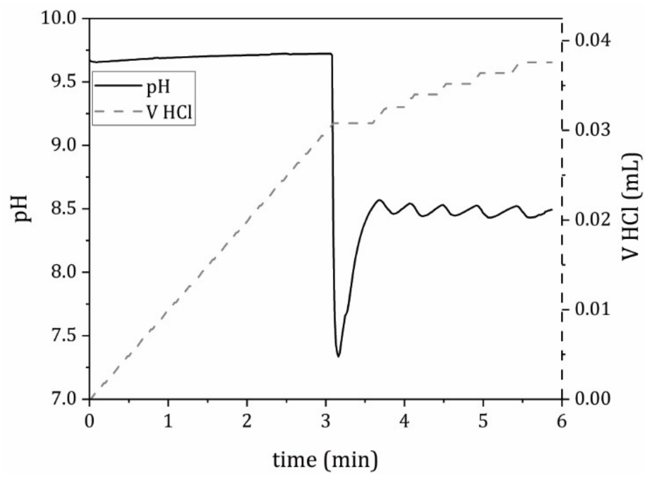

Figure S2. Acid titration after mixing GR with collagen and pAsp. The pH is monitored over time (black solid line) as HCl is added to the mixture (grey dashed line) to set the pH value to 8.5. The pH fluctuates around this value, but later pH measurements of an aged sample show that the pH is stable at about pH 8.6.

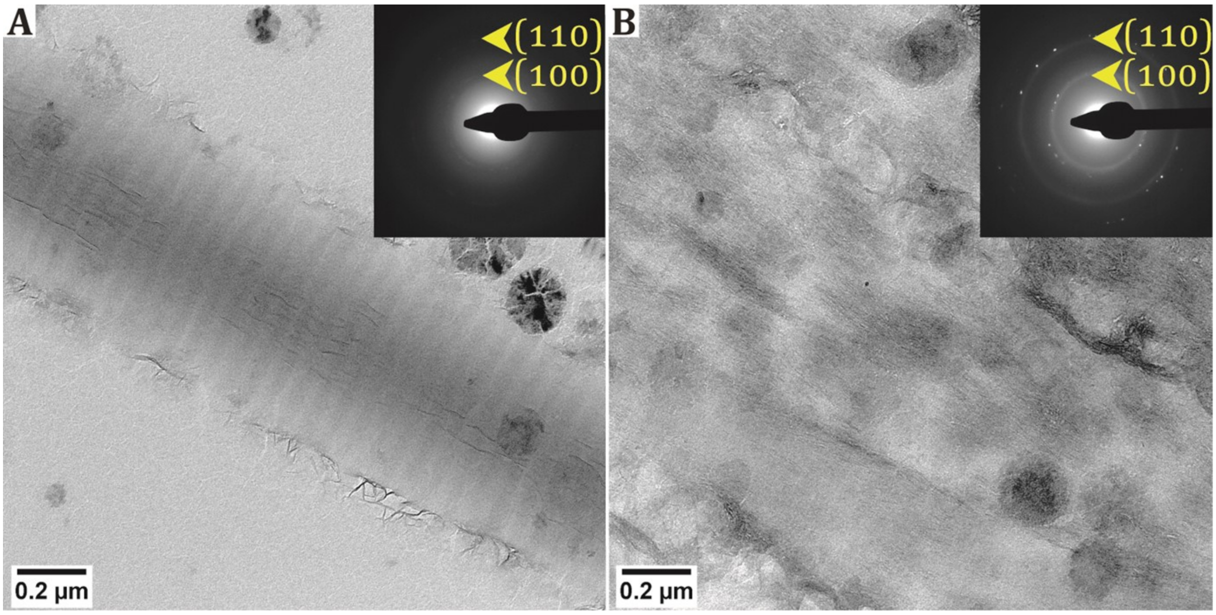

Figure S3. Collagen mineralization via the addition of $Fe(OH)_2$ after aging the reaction solution for 72 h. A, B. Dry-TEM image of the product. Insets: SAED of the corresponding images. In both cases, the SAED pattern matches with δ-FeOOH, which is possibly an oxidation product of $Fe(OH)_2$ upon exposure to air. No crystallographic alignment with the collagen is observed.

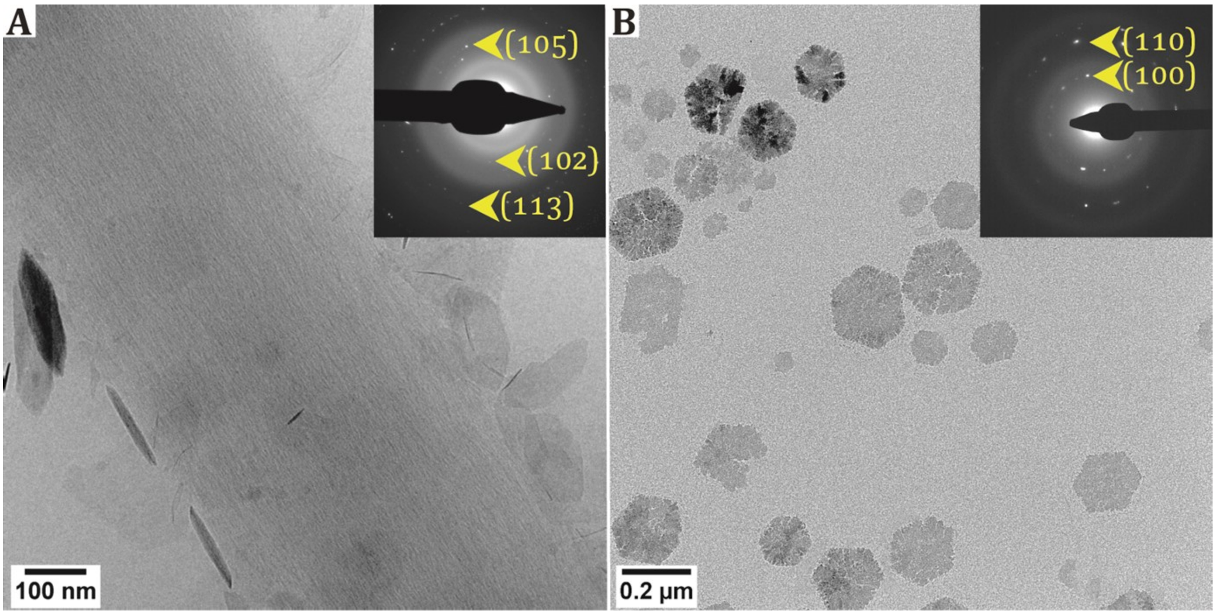

Figure S4. **Crystals outside the collagen in the mineralized sample. A.** Cryo-TEM image of a collagen fibril, with hexagonal crystals surrounding the fibril. Inset: SAED indicates that GR crystals are present outside the collagen. **B.** Dry-TEM image of the crystals outside the collagen. Inset: SAED indicates the formation of δ-FeOOH, which is possibly an oxidation product of GR.

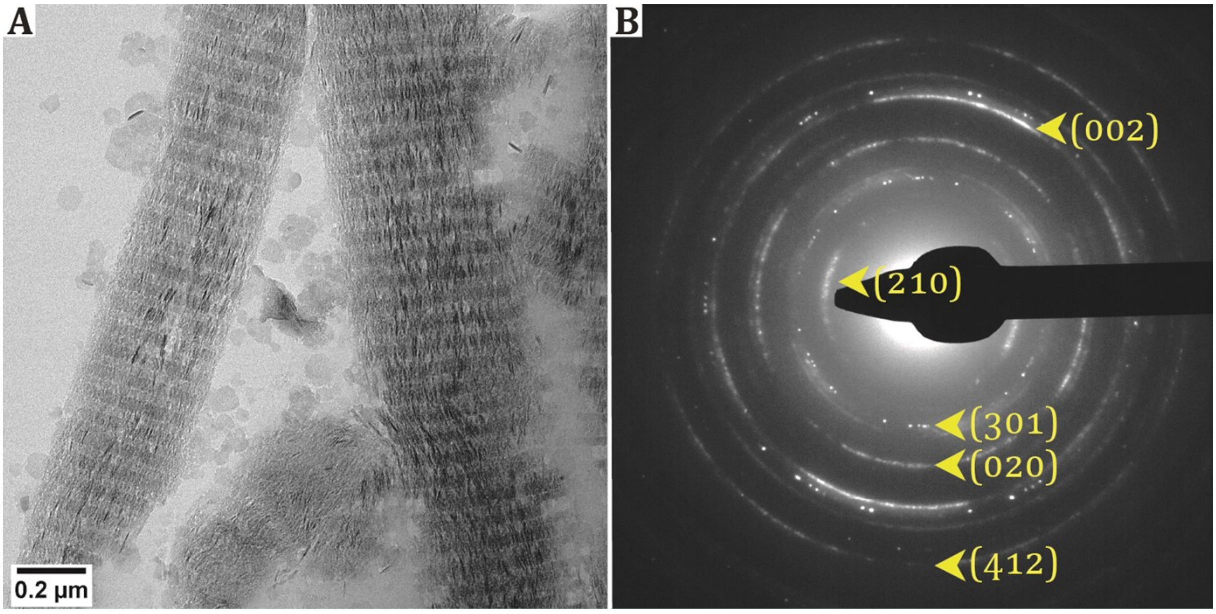

Figure S5. Mineralization of collagen via the addition of GR crystals to collagen and pAsp. In this case, the theoretical amount of base necessary for full conversion of all Fe^{2+} into $Fe(OH)_2$ was added. The pH was not adjusted. Although all precautions were taken, the possibility that the dispersion has been in contact with acid cannot be avoided completely, as the pH probe and stirrer bars are cleaned with acid on a regular basis. **A.** Dry TEM image of the product after aging the reaction solution for 72 h. **B.** SAED pattern corresponding to the image in **A**, indicating the formation of lepidocrocite, with the (002) crystal axis aligned with the collagen.

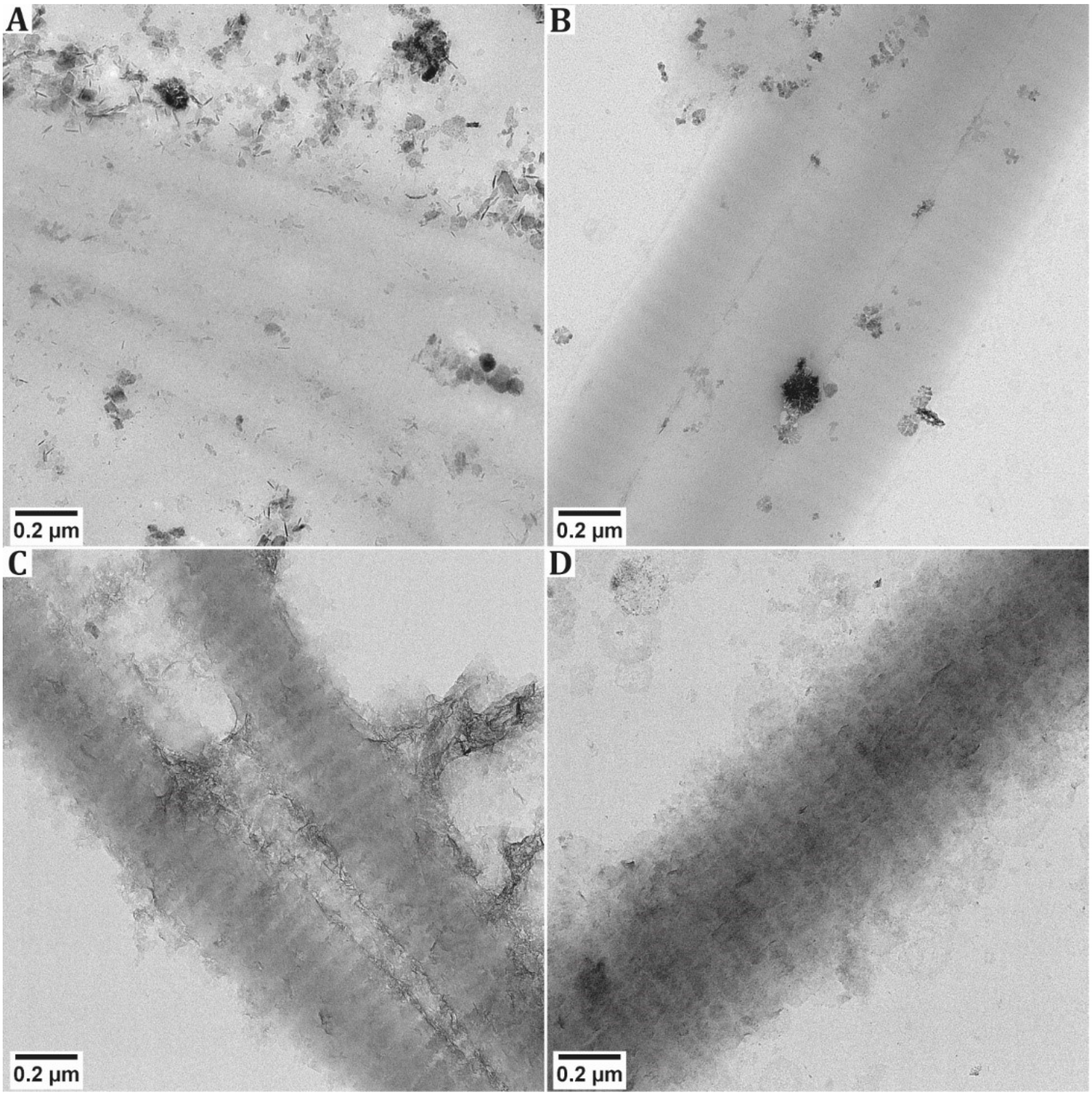

Figure S6. Oxidation of the reaction products in air. Dry TEM images of products exposed to air. **A, B, D.** Product after addition of $Fe(OH)_2$ to collagen and pAsp. For **B** and **D**, the pH of the mixture was adjusted to pH 8.5. For **A**, the pH was not adjusted after mixing of $Fe(OH)_2$ and collagen. **C.** Product after titration of a mixture of pAsp, collagen and $FeCl_2$ to pH 9. **A** and **B** were obtained from freshly made dispersions that were exposed to air for 1 h. **C** and **D** were aged inside the glovebox for several weeks first, after which the vials were removed from the glovebox and exposed to air for 3 h. Notably, **D** was obtained by exposing the same reaction dispersion from which the highly mineralized fibers were observed (Figure 2A, main text) to air.

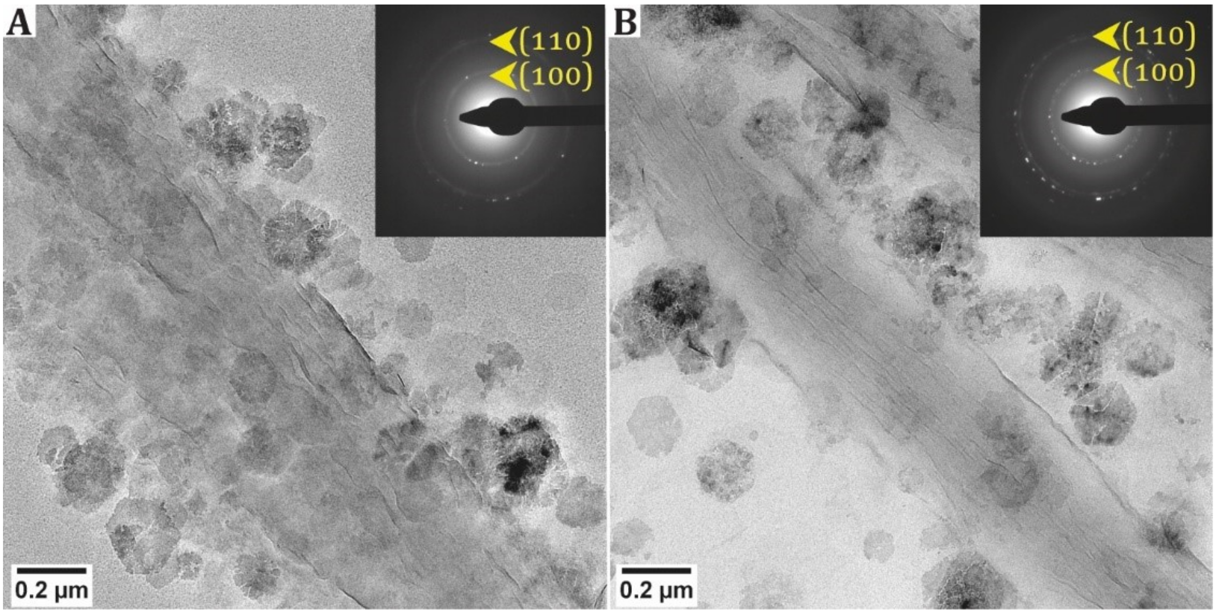

Figure S7. The effect of drying inside and outside the glovebox. The same reaction dispersion was used that yielded highly mineralized fibrils as demonstrated in Figure 2A (main text). Both grids were dried on top of a filter paper and subsequently imaged in dry TEM. **A.** The grid was removed from the glovebox after 5 min inside the glovebox and dried subsequently for another 30 min in air. **B.** The grid was removed from the glovebox after drying for 2.5 h. Insets: SAED corresponding to the image, both indicating the formation of δ-FeOOH, which is possibly an oxidation product of $Fe(OH)_2$.

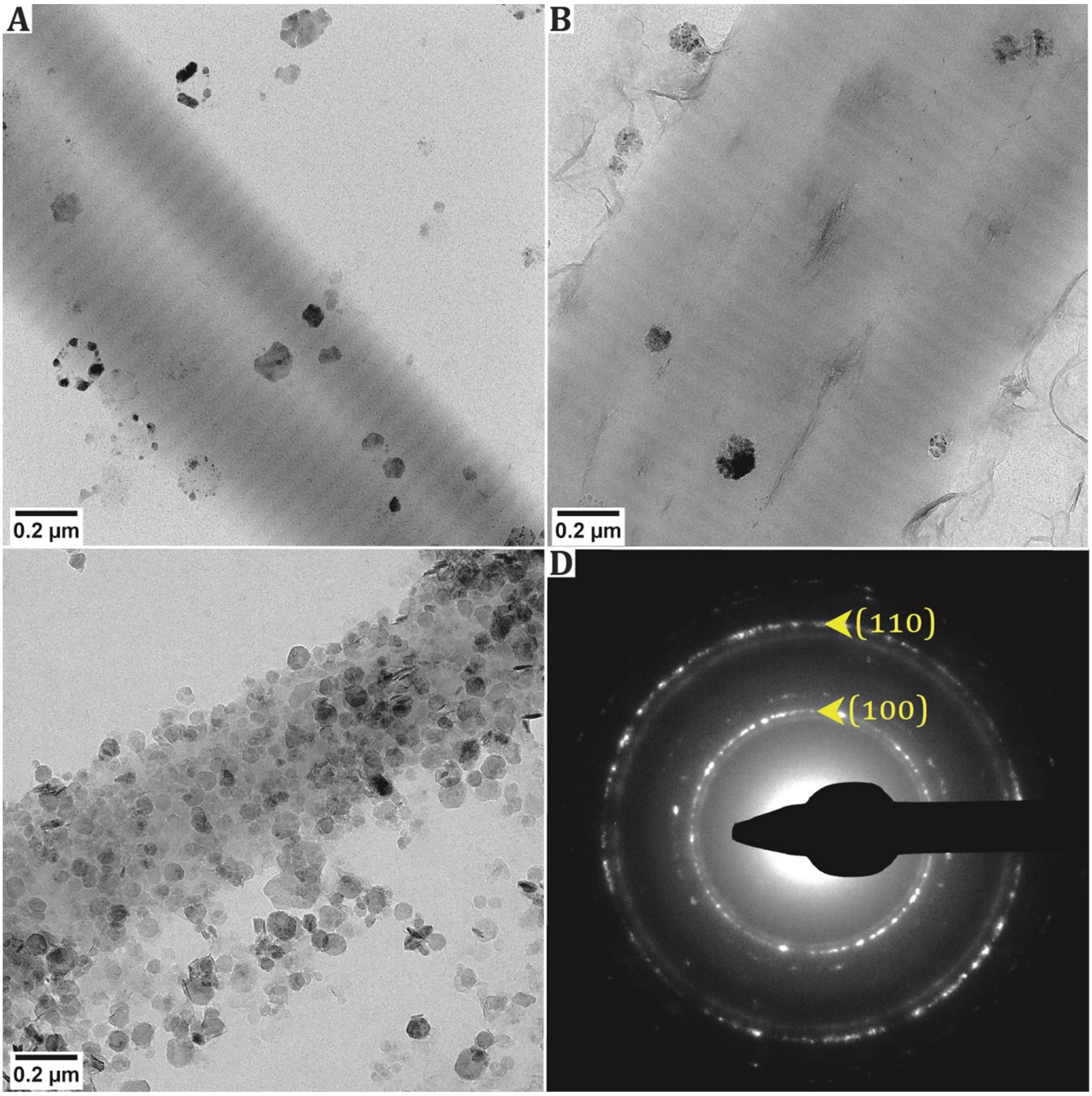

Figure S8. The effect of pAsp concentration. Dry TEM images of the product after **A.** performing the mineralization reaction with double pAsp concentration. **B**. performing the mineralization reaction with double pAsp and double Fe concentration and **C.** Performing the mineralization reaction in absence of pAsp. **D**. SAED corresponding to the TEM image in **C**, indicating the formation of green rust outside the collagen.

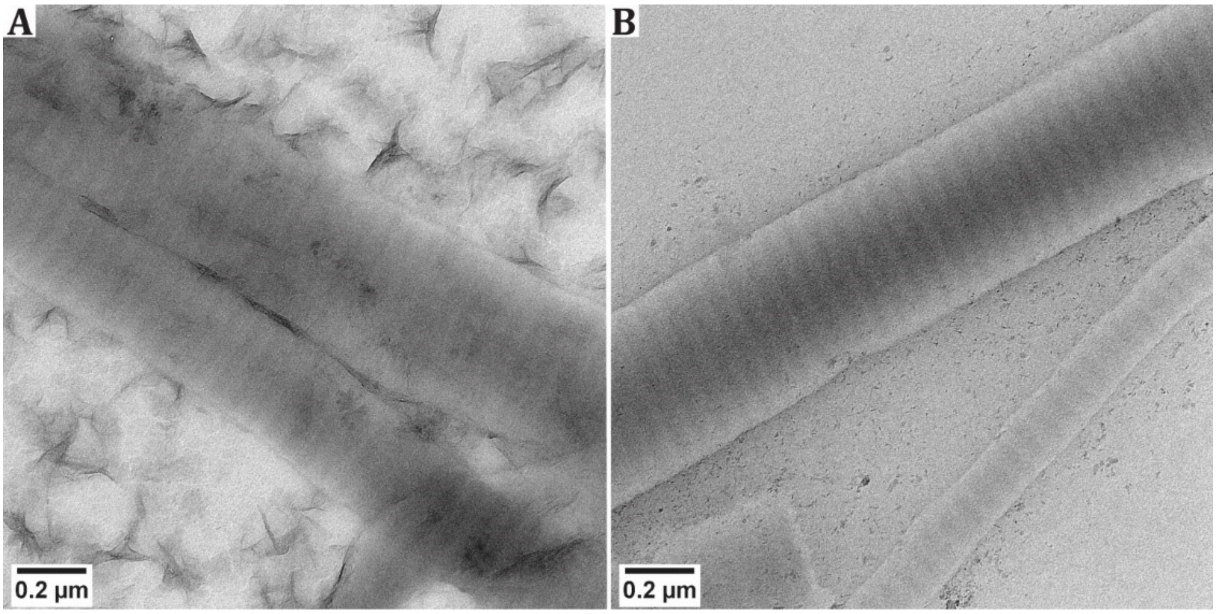

Figure S9. Adjusting the pH to 7.5 and 6 after mixing GR with collagen and pAsp. Dry TEM images of the product after adjusting the pH via addition of 0.5 M HCl to **A.** pH = 7.5 and **B.** pH = 6. In both cases, SAED did not give any signal. In **B**, all crystals seem to be dissolved.

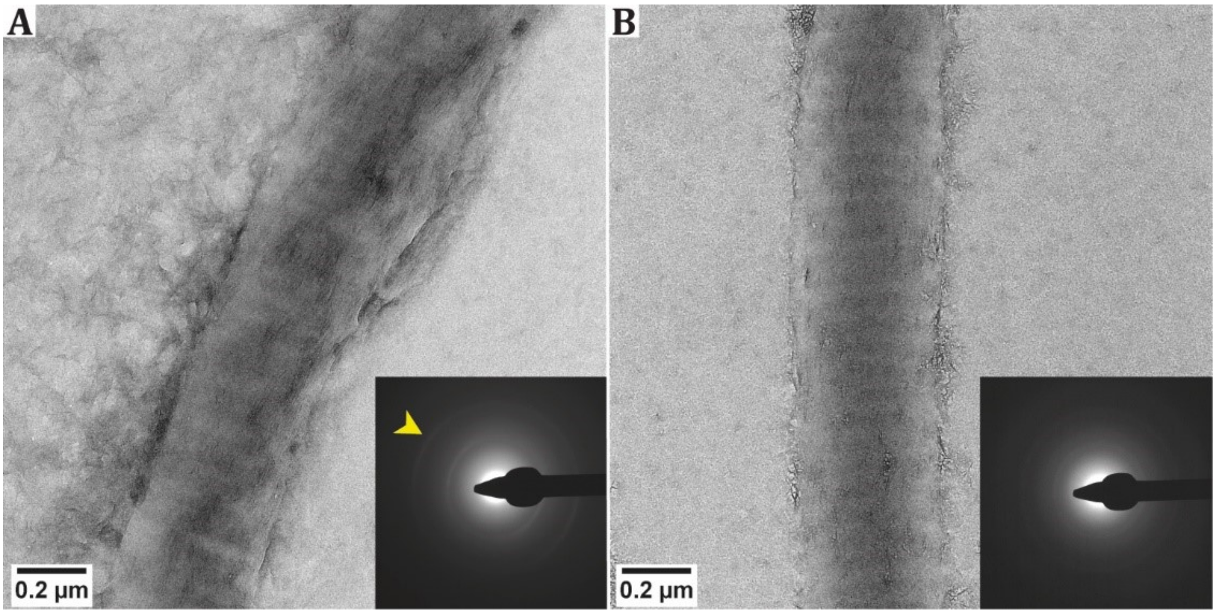

Figure S10. Mixing pAsp, collagen and $FeCl_2$ prior to titration with base. Dry TEM images of the product obtained by titrating 0.7 M KOH to the mixture of collagen, pAsp and $FeCl_2$ and aging for 72 h. Insets: SAED pattern corresponding to the TEM image. In **A** arcs are observed in the diffraction pattern (arrow), which could indicate the formation of intrafibrillar material, while the repetition of the experiment (**B**) does not indicate the formation of intrafibrillar crystals.

S3. Electron Tomography

The tilt series was recorded at a nominal magnification of 24k× and at a defocus of –1 µm. Alignment was performed in IMOD on the unbinned tilt series. The residual error mean obtained was 1.13, with a standard deviation of 0.87.

The tomogram was reconstructed using the Simultaneous Iterative Reconstructive Technique (SIRT) algorithm, with a standard Gaussian cutoff of 0.4 and a fall-off of 0.035. To obtain a reasonable balance between contrast and noise levels, 10 iterations were retained.

The electron tomography results are shown in **Figure 2B-C** (main text) and in **Figure S10**.

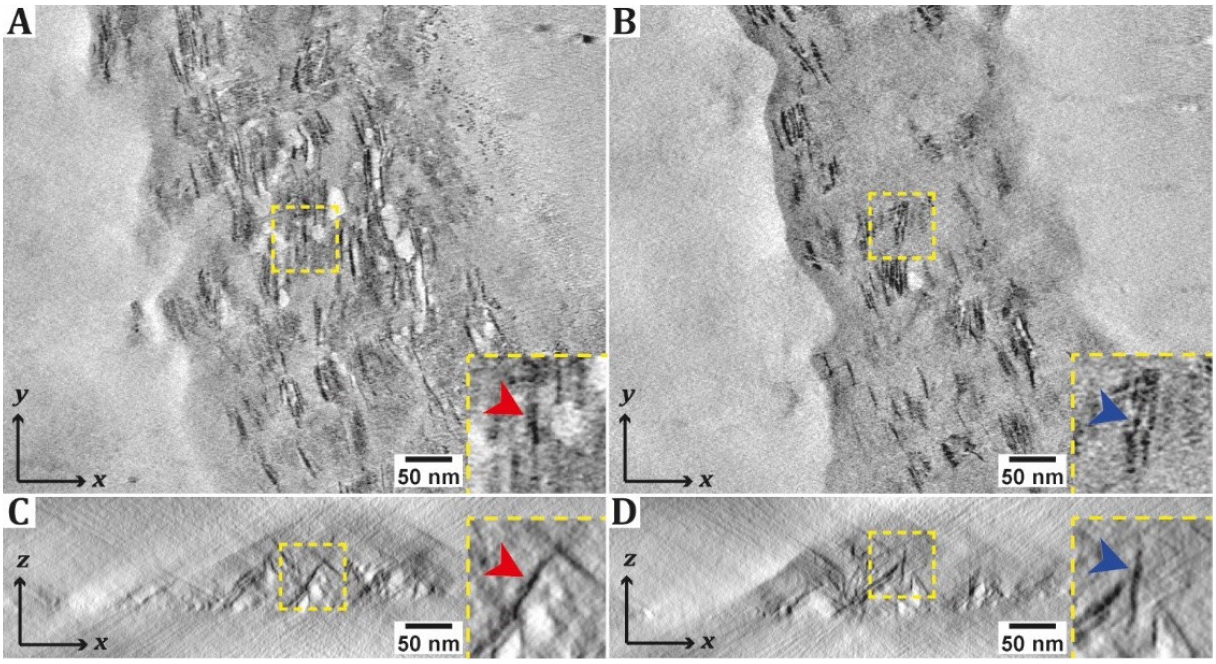

Figure S11. Electron tomography results of collagen fibrils mineralized via addition of GR crystals. A-B. Numerical cross-section (thickness: 0.5 nm) through the 3D ET reconstruction along the *xy*-plane. **C-D.** Numerical cross-section (thickness: 0.8 nm) through the 3D ET reconstruction along the *xz*-plane, corresponding to images **A** and **B**, respectively. Insets: magnified image of the crystals in the 3D-reconstructed volume. Arrows indicate the same crystal in both slices. Images are averaged over 3 slices to reduce noise.

S4. References

1. B. M. Oosterlaken, M. M. J. van Rijt, R. R. M. Joosten, P. H. H. Bomans, H. Friedrich, G. de With, *ACS Biomaterials Science & Engineering* 2021, DOI: 10.1021/acsbiomaterials.1c00416.
2. R. M. Cornell, U. Schwertmann, *The Iron Oxides*, Wiley-VCH Verlag GmbH&Co., Weinheim **2003**.
